# Molecular Alterations Associated with Acquired Drug Resistance during Combined Treatment with Encorafenib and Binimetinib in Melanoma Cell Lines

**DOI:** 10.3390/cancers13236058

**Published:** 2021-12-01

**Authors:** Vikas Patel, István Szász, Viktória Koroknai, Tímea Kiss, Margit Balázs

**Affiliations:** 1 Doctoral School of Health Sciences, University of Debrecen, 4032 Debrecen, Hungary; vikas.patel@med.unideb.hu; 2 MTA-DE Public Health Research Group, University of Debrecen, 4032 Debrecen, Hungary; szasz.istvan@med.unideb.hu (I.S.); koroknai.viktoria@med.unideb.hu (V.K.); 3 Department of Public Health and Epidemiology, Faculty of Medicine, University of Debrecen, 4032 Debrecen, Hungary; kiss.timea@med.unideb.hu

**Keywords:** malignant melanoma, *BRAF^V600E^*, combination of encorafenib and binimetinib, BRAF and MEK inhibitors, drug resistance, proteome array, RNAseq, drug holiday

## Abstract

**Simple Summary:**

Melanoma is the most aggressive, deadliest form of skin cancer. Combined BRAF-MEK inhibitor (BRAFi/MEKi) therapy was a breakthrough in the treatment of melanoma with BRAFV600-mutations. However, many patients frequently develop drug resistance to the combinatory treatment. The aim of our study was to characterize the molecular background behind acquired resistance to BRAFi/MEKi-s. After the successful development of resistant cell lines, we investigated the invasion properties, changes in gene and protein expressions, as well as the effect of the “drug holiday” of the resistant cell lines. Drug-resistant melanoma cells had a higher invasive potential and acquired a spindle-like structure, and many cancer-related proteins were overexpressed in the resistant cells. Furthermore, transcriptome analysis revealed that differentially expressed genes are functionally linked to a variety of biological functions that may lead to resistance to the inhibitors. These results may offer valuable insight into further understanding of BRAFi/MEKi resistance, as well as to the development of therapeutic tools to overcome drug resistance.

**Abstract:**

Combination treatment using BRAF/MEK inhibitors is a promising therapy for patients with advanced *BRAF^V600E/K^* mutant melanoma. However, acquired resistance largely limits the clinical efficacy of this drug combination. Identifying resistance mechanisms is essential to reach long-term, durable responses. During this study, we developed six melanoma cell lines with acquired resistance for BRAFi/MEKi treatment and defined the molecular alterations associated with drug resistance. We observed that the invasion of three resistant cell lines increased significantly compared to the sensitive cells. RNA-sequencing analysis revealed differentially expressed genes that were functionally linked to a variety of biological functions including epithelial-mesenchymal transition, the ROS pathway, and KRAS-signalling. Using proteome profiler array, several differentially expressed proteins were detected, which clustered into a unique pattern. Galectin showed increased expression in four resistant cell lines, being the highest in the WM1617^E+BRes^ cells. We also observed that the resistant cells behaved differently after the withdrawal of the inhibitors, five were not drug addicted at all and did not exhibit significantly increased lethality; however, the viability of one resistant cell line (WM1617^E+BRes^) decreased significantly. We have selected three resistant cell lines to investigate the protein expression changes after drug withdrawal. The expression patterns of CapG, Enolase 2, and osteopontin were similar in the resistant cells after ten days of “drug holiday”, but the Snail protein was only expressed in the WM1617^E+BRes^ cells, which showed a drug-dependent phenotype, and this might be associated with drug addiction. Our results highlight that melanoma cells use several types of resistance mechanisms involving the altered expression of different proteins to bypass drug treatment.

## 1. Introduction

Despite several advancements in the treatment of patients with malignant melanoma, this disease is still one of the major causes of skin cancer-associated death [1]. Activating mutations in the *BRAF* oncogene are the most widespread genetic alterations observed in melanoma, with an incidence of up to 40–60% [2,3]. The most frequent mutation is an amino acid change from valine to glutamic acid at the 600-position: *BRAFV^V600E^*. This mutation enhances the kinase activity of mitogen-activated protein kinase (MAPK) signalling ~500-fold and controls important cellular functions involving cell proliferation, differentiation, migration, survival, and angiogenesis [4]. Since 2011, targeted inhibition of the mutant BRAFV600E protein has been the most promising therapeutic target for patients with advanced stage or metastatic melanoma [5]. A panel of BRAF inhibitors (BRAFi-s) (vemurafenib, dabrafenib, encorafenib, etc.) and a series of MEK inhibitors (MEKi-s) (cobimetinib, trametinib, binimetinib, etc.) have revolutionized the treatment of melanoma patients [6]. Combinational treatment using encorafenib and binimetinib was approved by the US Food and Drug Administration (FDA) for melanoma patients with a *BRAF^V600E/K^* mutations in 2018 [6,7]. This inhibitor combination showed a high response rate, a favourable toxicity profile, and impressive progression-free survival (approximately 16.9 months compared to the ~9-month BRAFi monotherapy) in melanoma patients with BRAF mutations [6,8]. Notwithstanding the high success of the targeted therapies, acquired resistance to treatment developed in a large number melanoma patients [9,10]. Many recent studies have clarified the molecular background of acquired drug resistance and identified new therapeutic targets to improve patient survival, but unfortunately the mechanism of resistance remains unclear. It is well documented that acquired resistance is driven by *NRAS*, *MAP2K1*, and *MAP2K2* mutations, *BRAF^V600E^* gene mutation or amplification, and different mutations in genes in the PI3K pathway [11]. Studies have also shown that several protein alterations, such as those in EGFR, COT, cyclin D1, and PDGFR-β, are associated with BRAFi and MEKi resistance [11].

Our aim during the current study was to explore the molecular alterations associated with the treatment of the ENCO+ BINI inhibitor combination in *BRAF^V600E^* mutant melanoma cell line models. Sequentially, we investigated resistance-associated genomic and proteomic alterations in melanoma cells. To achieve our goal, we first established six melanoma cell lines that became resistant after continuous treatment with BRAFi/MEKi. We compared the differences in cell proliferation, protein, and mRNA expression between the drug-sensitive and drug-resistant melanoma cell lines and studied the consequence of the “drug holiday” on cell proliferation in the resistant cells. Proteome profiler analysis and RNA sequencing (RNA-Seq) transcriptome profiling analysis were performed to describe the possible alterations leading to the development of acquired resistance during encorafenib plus binimetinib treatment.

## 2. Materials and Methods

### 2.1. Cell lines and Culture Reagents

Melanoma cell lines were obtained from the Coriell Institute for Medical Research (Camden, NJ, USA). All cell lines were cultured under the same conditions as described before [12]. The BRAF and NRAS mutational status, the origin, and other characteristics of all melanoma cell lines are described in Table 1.

### 2.2. Establishment of Resistant Cell Lines

Resistant cell lines were generated through long-term high-dose treatment using increasing concentrations of ENCO+BINI in every passage as described by Szász et al. [12]. In brief, cells (70% confluence) were switched to drug-containing growth medium and allowed to grow for three months (5% CO_2_ at 37 °C). The concentration of the drug combination at the beginning of treatment was 1 nM for each cell line and increased up to 200 nM. To reach the maximum concentration of the drug combination, the time window varied between three and five months. Resistant cells were maintained in complete medium supplemented with a 200 nM inhibitor mixture. Comparisons of the different parameters (cell viability, invasive potential, protein expression, and transcriptome profile) between the sensitive and resistant cells were performed at the same passage number. Encorafenib (LGX818) and binimetinib (MEK162) were obtained from Selleck Chemicals LLC (Houston, TX, USA). Stock solutions were prepared in dimethyl sulfoxide (DMSO) and stored at −20 °C.

### 2.3. Cell Proliferation Assay

Cell proliferation was assessed using the WST-1 test (Sigma–Aldrich Inc., St. Louis, Missouri, USA) following the supplier’s protocol. Briefly, before drug treatment, melanoma cells (5 × 10^3^ cells/well/100 µL medium) were seeded into 96-well plates for 24 h. On the second day, the cells were treated with a drug combination of ENCO+BINI (1 µM) for 72 h. DMSO (solvent of the inhibitors) was added to the cells as a control. After 72 h, the cell viability was measured using the WST-1 assay (Epoch™ Microplate Spectrophotometer (BioTek Instruments, Inc., Winooski, VT, USA). The absorbance of the samples was measured at 440 nm. The control wavelength was set to 650 nm. The viability of the cells was defined by dividing the absorbance of the encorafenib plus binimetinib-treated cells by that of the DMSO-treated control cells (the absorbance of control cells was defined as 100%).

### 2.4. Drug Holiday Experiment

A drug holiday experiment was performed similarly as we published earlier [12]. Resistant cells were seeded (5 × 10^3^ cells/well/100 µL medium) into a 96-well plate (in triplicate) with culture medium containing 200 nM of ENCO+BINI inhibitors and incubated for 24 h. The next day, cells were switched to growth medium containing DMSO and were cultured for 72 h or were maintained in growth medium containing a 200 nM drug combination of ENCO+BINI for 72 h. After 72 h, 10% of WST-1 reagent was added directly to each well, and the cells were incubated for the next three hours at 37 °C. Absorbance was measured as described above. The absorbance of control cells (cells treated with DMSO) was considered 100%. Relative absorbance was transfigured into the relative percentage of viable cells.

### 2.5. In Vitro Invasion Assay

The invasive potential of the inhibitor (ENCO+BINI)-sensitive and -resistant melanoma cells was evaluated using BioCoat Matrigel Invasion Chambers (BD Biosciences, Bedford, MA, USA) as described before [13]. Shortly, for BRAFi/MEKi-sensitive cells, the upper part of the invasion chamber was filled with a serum-free cell suspension (500 µL), and growth medium with 10% FBS was used (as a chemoattractant) in the lower chamber. For BRAFi/MEKi-resistant cells, we filled the upper chamber with 500 μL of the melanoma cells (cells were kept in serum-free medium containing a 200 nM/ combination of the inhibitors). The culture medium in the lower chamber was supplemented with 10% FBS (as a chemoattractant) and a 200 nM combination of ENCO+BINI. The invaded cells were fixed with ice-cold methanol after 24-h incubation and stained with haematoxylin-eosin. Invasive cells were counted under microscope (200× magnification) in seven different areas, and cell numbers are displayed as the mean ± SD of three independent experiments.

### 2.6. Protein Expression Analysis

Protein expression analyses were performed as described in detail before [12]. Briefly, BRAFi/MEKi-sensitive and -resistant cells were cultured to ~nearly 80% confluence and washed gently (2×) using ice-cold PBS. One ml of RIPA Lysis and Extraction Buffer (Thermo Fisher Scientific Inc., Waltham, MA, USA) containing 20 µL protease and phosphatase inhibitor cocktail (Thermo Fisher Scientific Inc., Waltham, MA, USA) was added to each cell culture, and cells were removed from the tissue culture flask by applying a cell scraper blade (Thermo Fisher Scientific Inc., Waltham, MA, USA). Cell lysates were transported to microtubes, incubated on a rocking shaker (30 min, 4 °C), and centrifuged (13,000 rpm, 30 min, at 4 °C). The supernatants were transferred into new Eppendorf tubes, and the protein concentration was determined using the Bradford Protein Assay ((Bio-Rad Hungary Ltd., Budapest, Hungary) as described in the supplier’s instruction. The Proteome Profiler™ Human XL Oncology Array Kit was obtained from R&D Systems (R&D Systems, Inc., Minneapolis, MN, USA). Preparation of all the necessary reagents and the array procedure was performed according to the manufacturer’s detailed protocol The labelled proteins were detected and visualized using Chemi Reagent Mix (R&D Systems Inc., Minneapolis, MN, USA). The protein expressions (labeled spots on the membrane) were exposed using the Azure c300 Chemiluminescent Imaging System (Dublin, CA, USA) and were analysed using AzureSpot (Vesion: 2.2.167) software. The intensity of the positive control (reference spot) was considered 100%.

### 2.7. RNA Isolation

Total RNA preparation was carried out using an RNeasy Mini Kit (Qiagen GmbH, Hilden, Germany). RNA concentration was measured using a NanoDrop ND-1000 UV-Vis spectrophotometer. Only samples with ratios >1.8 (measured at 260/280 nm) were included in further analysis. An Agilent 2100 Bioanalyzer was used to evaluate sample quality before RNA sequencing (Agilent Technologies Inc., Santa Clara, CA, USA).

### 2.8. RNA Sequencing and Data Analyses

RNA-Seq and data analyses were performed as described previously [14]. To obtain global transcriptome data, high throughput mRNA sequencing analysis was performed on an Illumina sequencing platform. An Agilent BioAnalyzer with Eukaryotic Total RNA Nano Kit (Agilent Technologies, Waldbronn, Germany) was used for checking RNA integrity (RIN). RNA samples with integrity number > 7 were accepted for the library preparation process. mRNA-Seq libraries were prepared from total RNA using an Ultra II RNA Sample Prep kit (New England BioLabs Inc., Ipswich, MA, USA) according to the manufacturer’s protocol. Briefly, oligo-dT conjugated magnetic beads were used for mRNA enrichment, and then mRNAs were eluted and fragmented at 94 Celsius. Fragmented mRNAs were reverse-transcribed to single-stranded cDNA using random primers, and then double stranded cDNAs were generated. After end repair, A-tailing and the adapter ligation steps of the library preparation process were finished by amplification of adapter ligated fragments. Sequencing was performed on an Illumina NextSeq 500 instrument using single-end 75-cycle sequencing. The HISAT2 algorithm was used for alignment of raw sequencing reads to human reference genome version GRCh38. StrandNGS software (www.strand-ngs.com; accessed on 14 October 2020) was used for further statistical analysis. Aligned data were normalized using the DESeq algorithm, and then differentially expressed genes were determined by a moderated T-test with Bejamini-Hochberg FDR for multiple testing correction. A *p*-value 0.05 was considered statistically significant. Library preparations, sequencing, and primary data analysis were performed at Genomic Medicine and Bioinformatics Core Facility of the University of Debrecen, Hungary.

### 2.9. Gene Expression Analysis

Analysis of differentially expressed genes was carried out as described by Ahn et al. [15]. The following criteria were applied to determine significantly expressed genes in each sample: fold change ≥ 2 and *p*-value ≤ 0.05. The RNA-Seq data were deposited into the Gene Expression Omnibus (GEO) repository (http://www.ncbi.nlm.nih.gov/gds; accessed on 11 September 2021) under accession number GSE186108.

### 2.10. Gene Ontology Functional Analysis and Gene Set Enrichment Analysis (GSEA)

To gain mechanistic insight into the gene lists generated from the RNA-Seq data, a functional enrichment analysis was performed to identify the biological pathways more enriched in a gene list than would be expected by chance. The ToppFun tool ToppGene suite (https://toppgene.cchmc.org; accessed on 10 January 2021) was applied to find the functional enrichment of genes with at least a 2-fold change difference between the treated (resistant to BRAFi/MEKi) and control groups (sensitive to BRAFi/MEKi) based on GO pathways under default settings and a *p*-value cut-off of 0.05.

To identify gene sets from the Molecular Signatures Database (MSigDB) v7.2 (c5.all.v7.2.symbols.gmt), GSEA version 4.1.0 was used, which summarizes and represents specific well-defined biological states or processes and displays coherent expression values (www.gsea-msigdb.org; accessed on 1 October 2021).

### 2.11. Pathway Analysis

Significant pathways associated with specific gene expression signatures were identified using the EnrichR web-based application (http://amp.pharm.mssm.edu/Enrichr/#; accessed on 21 September 2021). Only significantly altered signalling pathways (*p*-value ≤ 0.05) were included in the analyses. Benjamini-Hochberg adjustment (FDR < 0.05) was applied as a cut-off, and pathways with ≥5 significantly differentially expressed genes were considered and used to define molecular pathways associated with the differentially expressed genes.

### 2.12. Quantitative Real-Time PCR

Quantitative real-time PCR (qRT-PCR) was used to define the relative mRNA expression of selected genes in five melanoma cell lines (WM983A, WM983B, WM278, WM1617, and WM902B and their BRAFi/MEKi resistant pairs) using a Light Cycler 480 Real-Time PCR System (Roche Diagnostics GmbH, Mannheim, Germany). cDNA synthesis was performed applying High-Capacity cDNA Reverse Transcription Kit (Life Technologies (Applied Biosystems, Waltham, MA, USA)) with random primers as described in the supplier’s protocol; 600 ng of total RNA for each reaction was used. SYBR Premix Ex Taq (Takara Holding Inc., Kyoto, Japan) was applied to carry out the qRT-PCR reaction. GAPDH (glyceraldehyde-3-phosphate dehydrogenase: Hs9999 9905_m1) was used as a reference gene, and the Livak method (2^-ddCT equation) was applied for the qRT-PCR data analyses [16]. The primer sequences for the selected genes are summarized in Supplementary Table S1. Environmental contamination was evaluated by including no template control (NTC) reactions.

### 2.13. Statistical Analysis

Statistical analysis was performed using SPSS (IBM SPSS 19.0, SPSS Inc., Chicago, IL, USA) and Graph Pad Prism 9 (Graph Pad Software Inc., San Diego, CA, USA) software. Pearson’s correlation coefficient was calculated to correlate the RNA-seq and qRT-PCR data. The cellular parameters were statistically analyzed using the Mann–Whitney–Wilcoxon test. Only a *p*-value < 0.05 was considered statistically significant. Data are presented as the average ± standard deviation (±SD) of at least three independent experiments. Error bars on the figures represent ±SD.

## 3. Results

### 3.1. Growth-Inhibitory Effect of the ENCO+BINI Combination on Melanoma Cell Lines and the Development of Resistant Cell Lines

**Figure 1:**
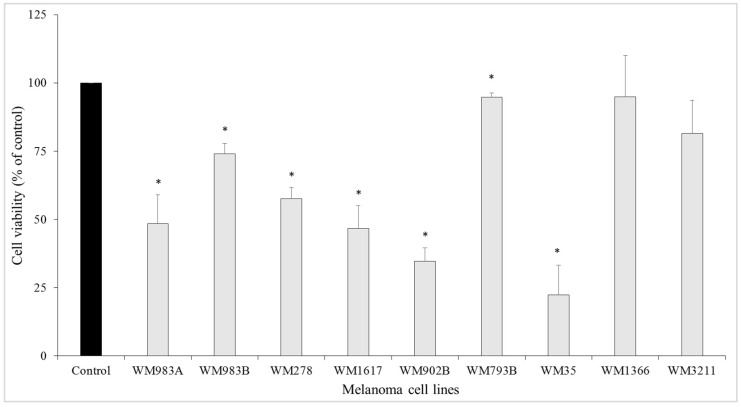
Growth-inhibitory effect of the combination treatment (ENCO+BINI) on melanoma cell lines. Melanoma cell lines were treated with a 1 µM drug mixture. After 72 h of incubation, cell viability was measured using a WST-1 assay. The data are presented as the mean ± SD of three independent experiments. The asterisks indicate statistically significant differences (Mann–Whitney–Wilcoxon test; * *p* < 0.05).

We investigated the growth-inhibitory effect of the BRAFi/MEKi combination treatment on nine melanoma cell lines. Seven cell lines carried the *BRAF^V600E^* mutation, one the *NRAS^Q61L^* mutation (WM1366), and one (WM3211) was wild type for both genes. The viability of melanoma cells was investigated using 1 µM drug mixture for 72 h. The combined BRAFi/MEKi treatment resulted in a significant (*p* < 0.05) decrease in cell viability in cell lines carrying the *BRAF^V600E^* mutation (Figure 1).

The most significant decrease (more than 30%) was observed in five cell lines (WM35, WM902B, WM1617, WM983A, and WM278). WM793B cells were sensitive to the treatment, but the viability of these cells decreased by less than 10%. In contrast, there was no significant decrease in cell viability in the *NRAS^Q61L^* mutant (WM1366) or *BRAF/NRAS* wild-type (WM3211) melanoma cell line.

**Figure 2:**
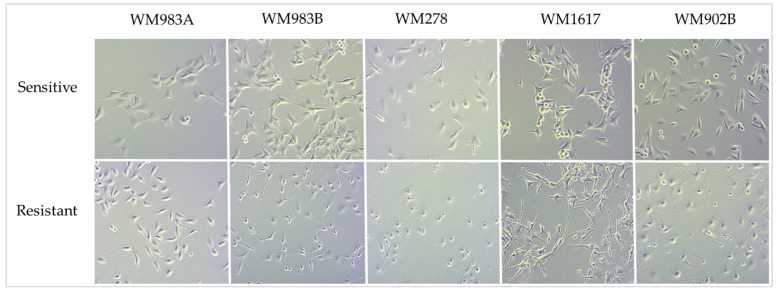
Photomicrographs of the ENCO+BINI-sensitive (**upper** panel) and ENCO+BINI-resistant (**lower** panel) melanoma cell lines. All images were captured at 100x magnification.

To establish resistant cell line variants during the ENCO+BINI treatment, the cells were treated continuously with increasing concentrations of the drug combination for 3–5 months, starting at 1 nM and increasing with every passage up to 200 nM. The morphology of the drug-sensitive cells differed from that of the ENCO+BINI-resistant cells, and the resistant cells mainly displayed an elongated phenotype (Figure 2).

**Figure 3:**
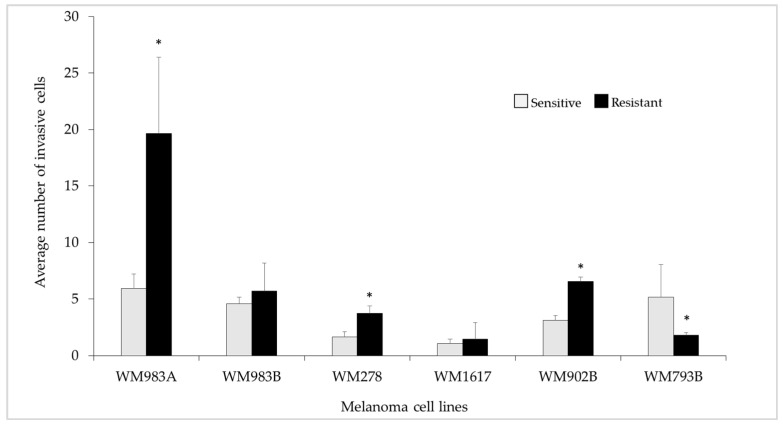
Invasive potential of the ENCO+BINI-resistant melanoma cell lines. Light grey columns correspond to the sensitive, and black columns represent the inhibitor-resistant cell lines. The data are presented as the mean ± standard deviation (±SD) of three independent experiments. The asterisks indicates a statistically significant difference (Mann–Whitney–Wilcoxon test; * *p* < 0.05).

We determined the invasive behaviour of the resistant cell lines and compared it to that of the sensitive cell lines using a Matrigel invasion assay, because morphological changes are often associated with increased invasive potential, and resistant cells often show epithelial-mesenchymal transition (EMT). Three drug-resistant cell lines (WM983A^E+BRes^, WM278^E+BRes^, and WM902B^E+BRes^) showed significantly enhanced invasive properties compared to their corresponding sensitive cell lines (Figure 3).

The invasive properties of two metastasis-derived resistant cell lines (WM983B^E+BRes^ and WM1617^E+BRes^) did not change significantly. However, the WM793B^E+BRes^ cells showed significantly decreased invasive potential compared to the sensitive cells.

### 3.2. Protein Array Analysis of the Parental and Resistant Cell Lines

**Figure 4:**
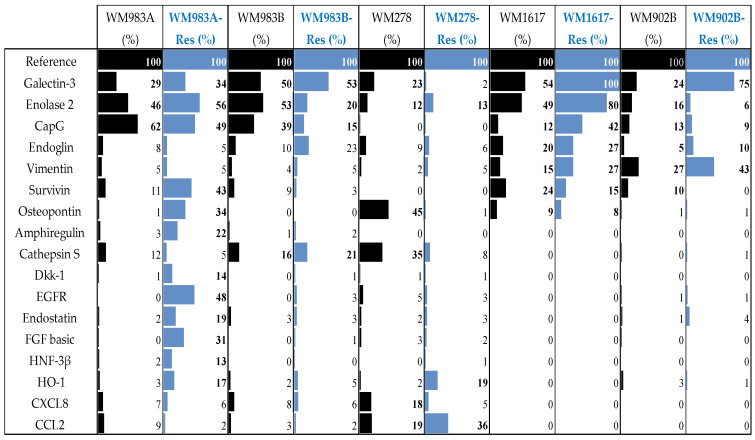
Protein expression profiles of BRAFi/MEKi-sensitive (black columns) and -resistant (blue columns) melanoma cell lines. Protein expressions were analysed using the Proteome Profiler Human XL Oncology Array. Proteins with detectable differences (>10%) in at least one cell line are shown. The intensity of the reference is displayed as 100%. Numbers in the columns indicate the protein expression as a percentage of the intensity of the reference spots on the array.

Oncology arrays (Proteome Profiler Human XL Oncology Array) were used to detect protein expression differences between BRAFi/MEKi-sensitive and -resistant melanoma cell lines. The array contained 84 cancer-related proteins. These analyses revealed numerous differentially expressed proteins in the resistant cell lines compared to their sensitive counterparts. However, we did not notice a similar expression pattern among the tested resistant cell lines (WM983A, WM983B, WM278, WM1617, and WM902B). Proteins with detectable differences (>10%) in at least one cell line revealed 17 differentially expressed proteins in the resistant cell lines; data are summarized in Figure 4.

A number of differentially expressed proteins (n = 12) were detected in the WM983A^E+BRes^ cells, including Survivin, Osteopontin, Amphiregulin, EGFR, FGF, and HO-1. Interestingly the expression of Galectin increased in four cell lines (WM983A^E+BRes^, WM983B^E+BRes^, WM1617^E+BRes^, and WM902B^E+BRes^), being the highest in the WM1617^E+BRes^ cells, but the expressions of other proteins were inconsistent.

### 3.3. Effect of Drug Withdrawal on the Viability and Protein Expression of the Brafi/Meki Resistant Melanoma Cell Lines

**Figure 5:**
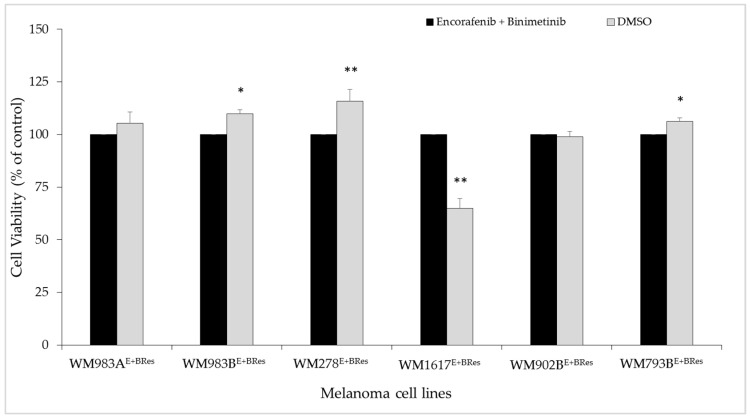
Effect of drug withdrawal on the viability of BRAFi/MEKi-resistant melanoma cell lines. The viability of the resistant cells after drug withdrawal (ENCO+BINI were removed, and DMSO was added for 72 h to the cell cultures: grey columns) was compared with that of cells that were treated continuously with the drugs (cells grown in the presence of 200 nM ENCO+BINI: black columns). The data are presented as the mean ± SD of three independent experiments. The asterisk indicates a statistically significant difference (Mann–Whitney–Wilcoxon test; * *p* < 0.05, ** *p* < 0.001).

Because acquired resistance can lead to drug dependency, we removed the drug mixture from the cell cultures and replaced it with the drug solvent (DMSO). Unexpectedly, we did not observe significantly decreased proliferation or cell death after 72 h of “drug holiday” in five cell lines (WM983A, WM983B, WM278, WM902B, and WM793B) compared to the control cell lines that were treated continuously with 200 nM ENCO+BINI, indicating that these cell lines are not addictive to the drug combination (Figure 5).

In addition, three resistant cell lines (WM983B^E+BRes^, WM278^E+BRes^, and WM793B^E+BRes^) exhibited significantly increased cell proliferation after 72 h of “drug holiday”, which indicates that these cells still experienced drug pressure. In contrast, the WM1617^E+BRes^ cell line behaved differently and showed significantly reduced cell proliferation (Figure 5)**.**

To determine whether drug dependence could be developed in resistant cells, we selected three cell lines (WM983A^E+BRes^, WM983B^E+BRes^, and WM1617^E+BRes^) and cultured the cells in the absence of the drug combination for 3 and 10 days and then measured the cell viability and protein expression changes and compared these to the control (cells were continuously treated with the inhibitor mixture).

**Figure 6:**
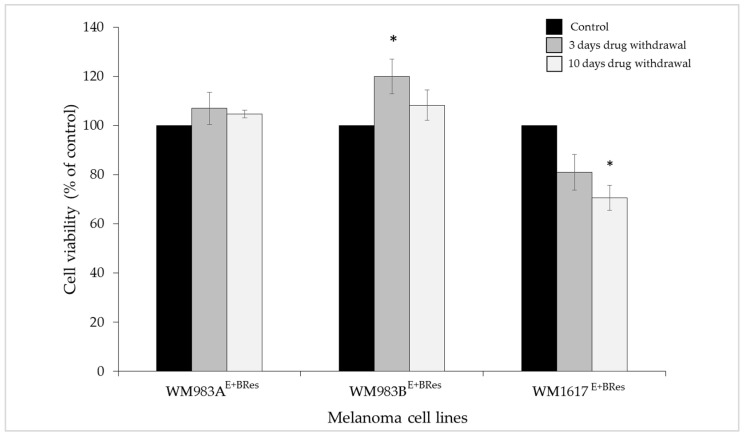
The effect of drug withdrawal on the viability of the resistant melanoma cell lines. Control cells were grown in the presence of 200 nM ENCO+BINI (black columns). Three days of drug withdrawal (cells grown in the presence DMSO for 3 days: dark grey columns), 10 days of drug withdrawal (cells grown in the presence DMSO for 10 days: light grey columns). The data are displayed as the mean ± SD of three independent experiments. The asterisk indicates a statistically significant difference (Mann–Whitney–Wilcoxon test; * *p* < 0.05).

Figure 6 clearly demonstrates that three days of drug withdrawal significantly increased cell viability in the WM983B^E+BRes^ cells, and a slight increase in the cell viability was observed in the WM983A^E+BRes^ cells. The viability of the cells did not decrease in these two cell lines even after 10 days of drug withdrawal; the viability of cells was higher than that of the continuously treated cells. In contrast, the viability of the WM1617^E+BRes^ cells decreased below 85% after three days of drug removal compared to the control cells, and a significant decrease was detected after 10 days of drug withdrawal (Figure 6).

**Figure 7:**
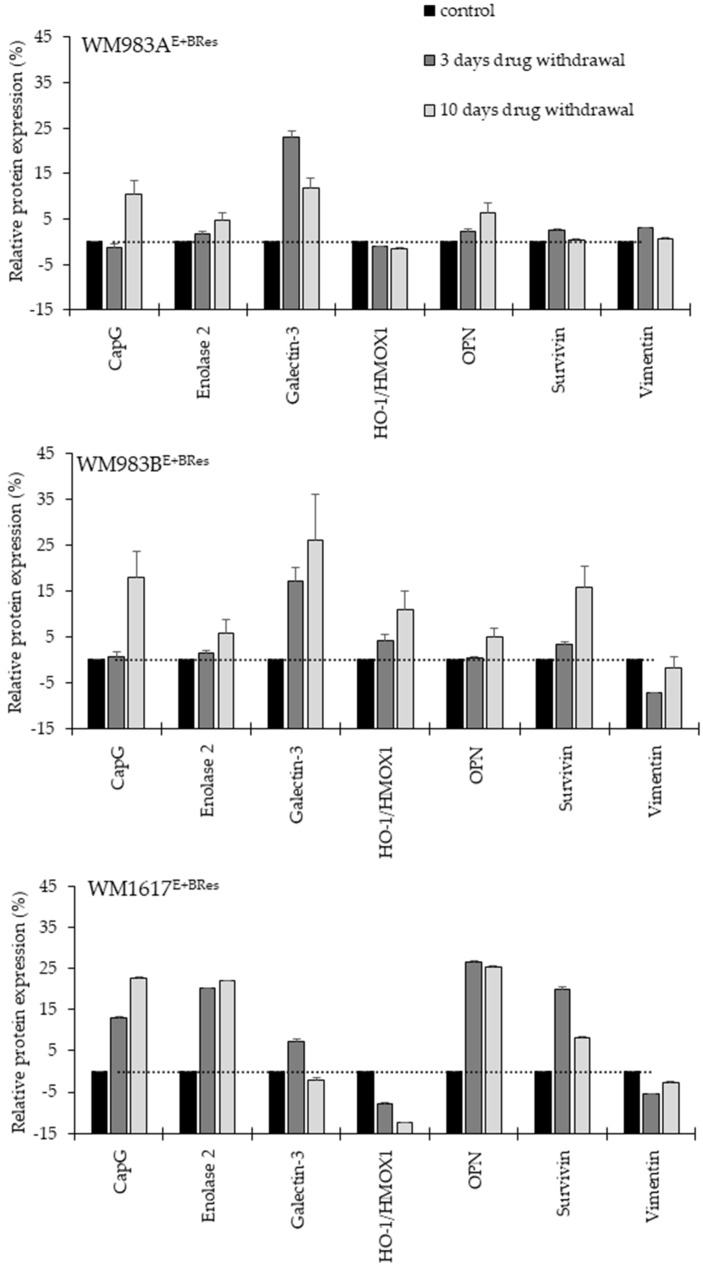
Protein expression changes of the BRAFi/MEKi-resistant melanoma cell lines after 3 and 10 days of drug withdrawal. Control cells were grown in the presence of the 200 nM inhibitor mixture (black columns). Drug withdrawal for 3 days (cells grown in the presence of DMSO: dark grey columns); drug withdrawal for 10 days drug withdrawal (cells grown in the presence DMSO: light grey columns). The data are displayed as the mean ± SD of two independent Protein Profiler Array experiments for each protein.

We also investigated the effect of the “drug holiday” on the protein expression changes using the same Proteome Profiler (Proteome Profiler Human XL Oncology Array) as we used before. After several days of the “drug holiday”, we detected seven proteins (CapG, Enolase 2, Galectin-3, HO-1/HMOX1, Osteopontin (OPN), Survivin, and Vimentin) whose expressions changed in all resistant cell lines following drug withdrawal (Figure 7).

The most extensive changes were seen after 10 days of drug withdrawal. The expressions of CapG, Enolase 2, and OPN proteins were increased in all cell lines. Galectin-3 also showed a notable increase in the WM983A^E+BRes^ and WM983B^E+BRes^ cell lines. The vimentin protein expression did not change after 10 days of drug removal in any cell line. The expression of survivin was variable in the different resistant cells. It should be noted that in addition to the co-expressed proteins, several other proteins were also highly expressed: Cathepsin S, EGFR, Endoglin CXCL8, and CCL20 were detected in the WM983B^E+BRes^ cells, and HIF-1α, Endoglin, P53, and Snail were detected in the WM1617^E+BRes^ cells (Supplementary Figure S1).

### 3.4. Identification of Differentially Expressed Genes in Resistant Melanoma Cell Lines Using RNA-Seq Analysis

We performed RNA-Seq analyses to determine gene expression patterns in BRAFi/MEKi-sensitive and BRAFi/MEKi-resistant melanoma cell lines. Gene expression analysis revealed a total of 1591 differentially expressed genes (1024 upregulated and 567 downregulated transcripts; fold change ≥ 2, *p*-value ≤ 0.05) in the resistant cell lines (Supplementary Table S2). The top 10 up-and downregulated genes are listed in Table 2.

**Figure 8:**
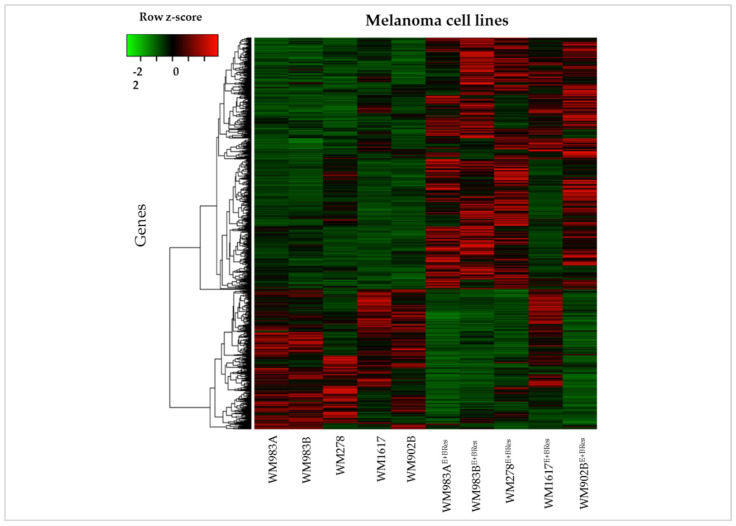
Unsupervised hierarchical clustering of the 1591 differentially expressed genes in BRAFi/MEKi-sensitive and -resistant melanoma cell lines. Cell lines are displayed vertically, and genes are displayed horizontally. The colour of each cell represents the median-adjusted expression value of each gene. Red represents increased gene expression, and green represents decreased gene expression.

Unsupervised hierarchical clustering of the 1591 differentially expressed genes distinguished between the BRAFi/MEKi-sensitive and -resistant melanoma cell lines (Figure 8). The drug-sensitive cell lines were grouped together. Interestingly, most of the genes that were downregulated in the inhibitor-sensitive cell lines were upregulated when acquiring the resistant phenotype, and the upregulated genes were downregulated. Similar gene expression patterns were observed in three resistant cell lines; only the WM1617^E+BRes^ cell line expression signature differed from the inhibitor-sensitive counterpart cells. The majority of the upregulated genes did not change during the development of the drug resistance in this cell line.

We applied qRT-PCR to confirm the transcriptional alterations of ten genes (CXCL12, COL5A1, ALPK2, ABCC3, CHST15, DMRT2, MRGPRX4, TRIM51, VEPH1, and GJB1). Seven of the ten genes examined (CXCL12, COL5A1, ABCC3, CHST15, DMRT2, MRGPRX4, and VEPH1) exhibited the same direction of gene expression in the parental and resistant cell lines.

The top 30 downregulated genes are listed in Supplementary Table S3 and the top 30 upregulated are listed in Supplementary Table S4 for each cell line.

### 3.5. GSEA of Differentially Expressed Genes

Using GSEA, we found several ontology gene sets that were significantly enriched, including regulation of cell proliferation, biological adhesion, and regulation of cell death, response to drug, vasculature development, regulation of cell development, and regulation of chromosome organization (*p* ≤ 0.05). The gene sets that correlated with drug resistance are displayed in Table 3.

**Figure 9:**
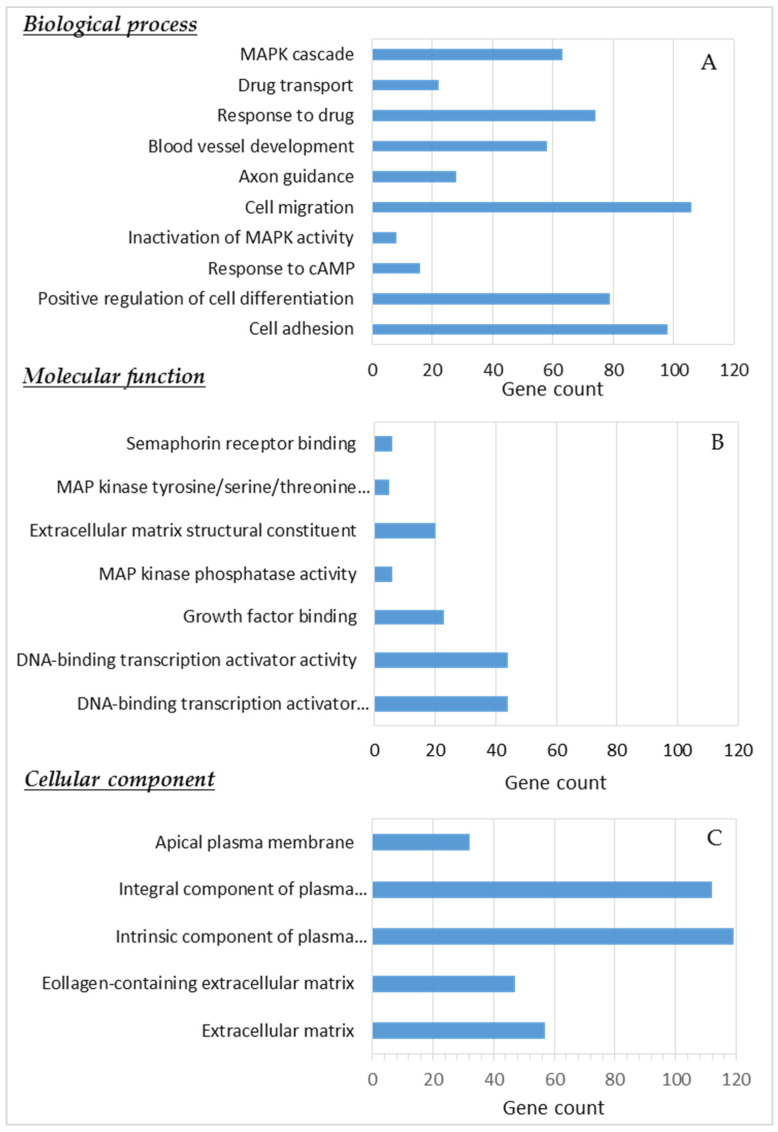
Gene Ontology analysis ((**A**) biological processes, (**B**) molecular functions, and (**C**) cellular components) of differentially expressed genes in ENCO+BINI-resistant cell lines. Significant differentially expressed genes with a fold change ≥ 2 and *p*-value ≤ 0.05 were analysed to determine the enriched Gene Ontology terms.

GO analysis of the differentially expressed genes revealed numerous significantly (*p*-value ≤ 0.001) enriched GO terms grouped by biological processes (n = 194), molecular functions (n = 9), and cellular components (n = 6); these included cell adhesion, cell migration, axon guidance, response to drug, regulation of the MAPK cascade, MAP kinase tyrosine/serine/threonine phosphatase activity, and the extracellular matrix (ECM) (Figure 9).

### 3.6. Pathway Analysis of Differentially Expressed Genes

To further understand the involved pathways and biological functions during the development of resistance, we performed pathway analysis using MSigDB. Based on this analysis, we observed that the differentially expressed genes were associated with a wide range of molecular pathways. The significantly altered pathways (those including at least five upregulated genes) were functionally associated with the following: ATF-2 transcription factor network, AP-1 transcription factor network, EMT, TNF-alpha signalling via NF-kB, and reactive oxygen species (ROS) pathway (Table 4). On the other hand, the downregulated genes were associated with the following: KRAS signalling, TNF-alpha signalling via NF-kB, inflammatory response, IL-2/STAT5 signalling, coagulation, and early oestrogen response. EMT is a crucial cellular process that promotes metastasis and is often associated with drug resistance. In line with this, we found that the upregulated genes were significantly associated with EMT.

**Figure 10:**
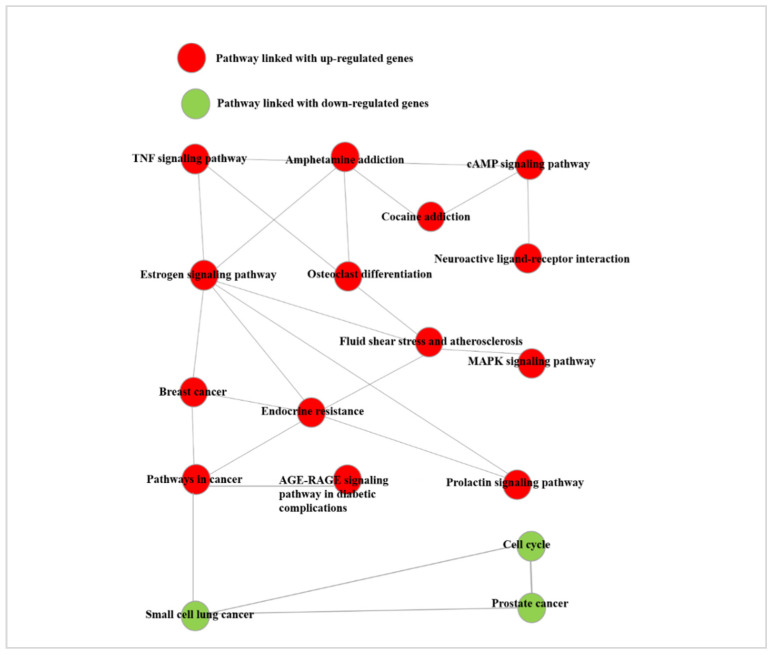
Interactions between the different signalling pathways (using NetworkAnalyst 3.0). Red dots represent upregulated pathways, and green dots represent downregulated pathways.

To further understand how the identified molecular pathways link to each other, a visual pathway network analysis was carried out (NetworkAnalyst 3.0). The interaction between key significant KEGG pathways was displayed. The approach was established on the notion that two pathways are linked if they share a certain fraction of genes (Figure 10). The majority of cancer-related pathways were upregulated, including the TNF signalling pathways, Estrogen signalling, and MAPK signalling pathways, and the cell cycle pathway was downregulated. Both groups of pathways were interconnected with pathways related to cancer. These data indicate that screening for DEGs and molecular pathways associated with acquired resistance in melanoma using integrated analyses could help us understand the molecular mechanism underlying the development of resistance, providing effective targets for the successful treatment of melanoma.

## 4. Discussion

Since the discovery of the *BRAF^V600^* mutations in malignant melanoma, the development of new drugs, including effective small molecule inhibitors of the MAPK signalling pathway, antibodies targeting immune checkpoint inhibitors including cytotoxic T-lymphocyte-associated antigen 4 (CTLA-4), programmed cell death (PD)-1, and PD-ligand 1 (PD-L1), has expanded dramatically [17,18]. Recently, the FDA approved new combinatorial treatments including atezolizumab (PD-L1 inhibitor) in combination with cobimetinib and vemurafenib for patients with *BRAF^V600^* mutation-positive advanced stage melanoma [19]. However, melanoma recurrence was diagnosed in most of the patients after the initial response. The combination of BRAF plus MEK inhibitors provides inspiring treatment options as a targeted therapy for patients with BRAF-mutated melanoma, with an improved overall response [20]. ENCO+BINI combination therapy was approved in 2018, and this treatment possibility has markedly enhanced therapy efficacy and tolerability compared to monotherapies [21]. These new targeted drug combinations have greatly improved the prognoses of patients with advanced and metastatic melanoma; however, regrettably, acquired resistance to most of these drugs limits the number of patients with long-lasting responses. Recently, a large number of investigations have focused on identifying the molecular alterations leading to drug resistance, but regrettably, the underlying mechanisms of this process remain unclear. It is a very urgent need to identify common, resistance-associated molecular targets in melanoma cells that will help us to discover more effective treatment possibilities for this aggressive cancer; therefore, testing all promising and effective treatment options is essential. The novelty of our study is that we were able to develop encorafenib plus binimetinib-resistant melanoma cell lines after three to five months of continuous BRAFi/MEKi treatment and compared the gene and protein expression differences between the drug-sensitive and drug-resistant cells. Based on our results, we highlight the molecular changes that arise during the evolution of acquired resistance. In contrast to monotherapy [12], we also observed that intermittent drug (combination of ENCO+BINI) dosing might not be beneficial for melanoma patients with a BRAF mutation.

During the development of the BRAFi/MEKi-resistant cell lines, we observed morphological changes of the resistant cells. The phenotype of these cells was dramatically changed compared to the BRAFi/MEKi-sensitive cells. Resistant cells were elongated and spindle shaped, and the morphology of only one cell line (WM1617^E+BRes^) was different. Similar to our observations, morphological changes were also noted by Dratkiewicz et al., and Szász et al. [12,22]. In addition to an altered morphology, drug-resistant cells frequently exhibit greater invasive potential. Similarly, we also observed that, with the exception of one cell line (WM793B^E+BRes^), the resistant cell lines had higher invasive potential than the original, sensitive cell lines. Additionally, using transcriptome analysis, we observed that numerous invasive markers, including MMP2, were substantially elevated (fold change = 3.135, *p*-value = 0.009) in the resistant cell lines [23,24].

From a clinical point of view, discontinuing targeted therapy is a promising treatment strategy to prevent the development of drug resistance. A number of preclinical and clinical investigations have pointed out that drug dependency on the therapeutic drug can develop in drug-resistant cells and suggested that periodic treatment may be clinically beneficial [12,25,26]. However, the newly published preclinical and clinical studies have failed to establish the benefits of intermittent dosing, and a “drug holiday” has become very controversial in terms of therapeutic improvement [26,27]. Similar to these findings, except for one cell line, we also observed that BRAFi/MEKi-resistant cells were not drug addicted and did not exhibit significantly increased lethality following the “drug holiday” [28]. Taken together, our findings indicate that intermittent dosing may not increase the effectiveness of routine treatments; nonetheless, intermittent dosing needs further validation.

Several molecular changes associated with drug resistance have previously been found; these include overexpression of EGFR, PDGFR, HGF, IGF, CRAF, COT, and MITF and downregulation of STAG2 or STAG3 [11,17]. During our earlier investigation, we found that BRAFi monotherapy resistance was associated with specific cancer-related proteins, as detected using the Proteome Profiler Human XL Oncology Array [12]. We extended our present study to identify particular protein expression patterns linked to acquired resistance. Our protein expression analysis revealed several differentially expressed proteins in the ENCO+BINI-resistant cell lines. Based on the protein array data, the differentially expressed proteins were clustered into a unique pattern. Of the 17 differentially expressed proteins, 13 were altered in the WM983A-resistant cell line. In this cell line, the high expression difference was associated with the Enolase, CapG, Survivin, and EGFR proteins. EGFR, which is associated with BRAFi resistance, was differentially expressed only in this cell line. Galectin showed increased expression in four of the five resistant cell lines; its expression was highest in the WM1617 ^E+BRes^ cell line (100%), followed by the WW902B^E+BRes^ cell line (75%). In the other two cell lines, the expression differences were less than 10%. Galectin-3 is involved in many different biological functions, including cell adhesion, cell activation, the cell cycle, apoptosis, cell growth, and differentiation as reported by Mourad-Zeidan et al. [29]. Galectin-3 expression is positively correlated with the metastatic potential of human melanoma cell lines; it plays an important role in cell–matrix adhesion during melanoma progression [30]. The anti-apoptotic role of galectin-3 in breast cancer contributes to resistance to chemotherapeutic agents and might have an effect during the development of acquired resistance in melanoma [31]. Enolase was expressed in all sensitive and resistant cell lines, but the direction of expression changes was not uniform. In two resistant cell lines (WM983B^E+BRes^ and WM1617^E+BRes^), its expression increased, and in all other cell lines, its expression decreased. Furthermore, Enolase 2 (Gamma-enolase) and CapG were expressed in at least two cell lines with a greater than 10% difference, and the expression of these proteins varied greatly across cell lines. Interestingly, Enolase 2 and CapG are used as tumour markers in the diagnosis and prognosis of several cancer types, and both proteins have been associated with cell proliferation, invasion, migration, and metastatic capacity in several types of cancer, including melanoma [32,33]. The high variability in protein expression between the resistant cell lines indicates the necessity for more personalized treatment.

EMT is a complex mechanism that enables tumour cells to switch from the epithelial to the mesenchymal phenotype, and this transition allows cells to migrate from the primary site [34]. Vimentin is a well-known marker of EMT, and this protein was differentially expressed in four resistant cell lines, and noticeably high expression were detected in two (WM1617^E+BRes^ and WM902^E+BRes^) in which galectin-3 was also highly expressed. The enhanced expression of the Vimentin protein in resistant cell lines was consistent with the findings of Molnár et al. [35].

The effect of continuous versus intermittent dosing of drugs is highly controversial regarding patient survival; only limited data are available about which molecular pathways are dominant in this phenomenon [27,36,37]. To investigate whether BRAFi/MEKi resistance is associated with drug dependence, we determined the changes in cell viability and protein expression of the “drug holiday”. It was an important experiment, because the effect and the mechanism of drug withdrawal remain unknown. Our observation was not consistent for the three melanoma cell lines tested. We observed typical drug dependence in one cell line (WM1617^E+BRes^); in these cells, drug withdrawal was associated with significantly decreased cell proliferation, indicating that the cells became addicted to targeted therapy, exposing potential therapeutic vulnerabilities and highlighting that intermittent dosing could potentially be beneficial for therapeutic gains for melanoma patients [38] and can lead to clinical benefits, including the regression of tumours and can enhance the survival of patients [39]. In contrast, the other two resistant cell lines (WM983A^E+BRes^ and WM983B^E+BRes^) responded with elevated cell proliferation during the “drug holiday”. On the other hand, drug withdrawal resulted in protein expression changes in all three cell lines. Using the proteome profiler, we identified seven proteins (CapG, enolase 2, galectin-3, HO-1/HMOX1, OPN, survivin, and vimentin) with altered expression after the removal of the inhibitors. Some of these proteins are well-known players in drug resistance [12,40,41,42,43]. However, this is the first study that highlights the co-expression changes of proteins related to the “drug holiday” in BARFi/MEKi-resistant melanoma cells.

Several studies using human melanoma cell lines have reported differentially expressed genes between BRAFi-sensitive and BRAFi-resistant cells [15,44]. However, little is known about the genes that are linked to acquired resistance to the combinatorial treatment with BRAFi/MEKi. Using RNA-Seq analysis, we found 1591 differentially expressed transcripts (1024 upregulated- and 567 downregulated genes). The top 10 upregulated genes were *CXCL12*, *COL5A1*, *ALPK2*, *ABCC3*, *CHST15*, *RP11-326A19.5*, *LAMA5*, *SAMD11*, *RP11-54O7.3*, and *HHI PL2*. Pathway analysis revealed that the upregulated genes were significantly associated with the ATF-2 transcription factor network, EMT, and ROS. The ECM component COL5A1 has already been determined to be associated with BRAFi resistance in melanoma [45]. The other genes among the top ten upregulated genes are also associated with melanoma progression and metastasis formation through different signalling pathways; for example, *ALPK2* is involved in cancer by regulating the cell cycle and DNA repair [46], and *HHIPL2*-has been linked to Hedgehog signalling in gastric cancer [47]. Moreover, overexpression of *SAMD11* is associated with radioresistance in oesophageal cancer cells [48]. The ATP-binding cassette (ABC) superfamily of active transporters is a well-known marker and potential target for multidrug-resistant cancers and is upregulated in drug-resistant cell lines [49]. Similarly, ATP Binding Cassette Subfamily C Member 3 (ABCC3) was shown to be significantly upregulated in our resistant melanoma cell lines compared to our sensitive melanoma cell lines. However, until now, these upregulated genes have not been linked to BRAFi/MEKi resistance, which enhances the novelty of the present study.

The top 10 downregulated genes were *DMRT2*, *MRGPRX4*, *TRIM51*, *CTD-2207A17.1*, *RP4-718J7.4*, *VEPH1*, *RP11-459E5.1*, *GJB1*, *ART3*, and *FABP7.* Pathway analysis discovered that the downregulated genes were associated with KRAS and IL-2/STAT5 signalling. These genes have earlier been described to play crucial roles in cell migration and metastasis formation, and all have been implicated in tumorigenesis through different pathways; for example, *DMRT* and *FABP7* are involved in EMT [50,51], and *VEPH1* suppresses vascularization by inhibiting AKT activation [52]. Additionally, elevated *VEPH1* expression suppresses EMT and invasion through TGF signalling pathway inhibition [53]. Similarly, this gene was substantially downregulated in our resistant cell lines.

Long non-coding RNAs (lncRNAs) regulate numerous biological processes in cancers through different molecular mechanisms and can be used as potential markers for monitoring therapeutic responses [54]. Emerging evidence suggests that the expression of lncRNAs is frequently associated with human cancer [54]. Similarly, our analysis revealed novel lncRNA transcripts including *RP11-326A19.5*, *RP11-459E5.1*, and *RP4-718J7.4*. The *RP11-326A19.5* transcript was upregulated in the developed resistant cell lines, whereas *RP11-459E5.1* and *RP4-718J7.4* were downregulated. Additionally, it was reported previously that some lncRNAs (*RP11-326A19.5* and *RP11-459E5.1*) are functionally involved in tumorigenesis and drug responses [55,56]. Furthermore, *RP4-718J7.4* is associated with inflammation and antibiotic resistance [57,58]. However, these transcripts are not well documented in the field of melanoma or in BRAFi resistance. The present study is the first to suggest that these long non-coding gene transcripts might play a role in BRAFi resistance, but there are no available data on the involvement of these genes during the development of acquired BRAFi/MEKi resistance.

## 5. Conclusions

Our current data offer the first insight into differentially expressed genes and provide protein expression patterns that are associated with a BRAFi/MEKi-resistant phenotype in melanoma cell lines. Our findings facilitate a more thorough understanding of the development of the complex mechanisms leading to acquired resistance during combined treatment in BRAF-mutant melanoma. However, further studies are needed to identify the key molecules and signalling pathways responsible for therapeutic escape during BRAFi/MEKi treatment and to prevent the initiation of acquired drug resistance in melanoma.

## Figures and Tables

**Table 1:** Characteristics of human melanoma cell lines.

Cell Line.	Sex/Age (Years)	Origin ^a^	Growth Phase ^b^		BRAF	NRAS
				Type ^c^	Mutation	Mutation
WM983A ^p1^	M/54	Primary	VGP	NM	V600E	wt
WM983B ^p2^	-	Metastasis	-	-	V600E	wt
WM278 ^m1^	F/62	Primary	VGP	NM	V600E	wt
WM1617 ^m2^	F/62	Metastasis	-	-	V600E	wt
WM902B	F	Primary	VGP	SSM	V600E	wt
WM793B	M/37	Primary	RGP/VGP	SSM	V600E	wt
WM35	F/24	Primary	RGP/VGP	SSM	V600E	wt
WM1366	M/79	Primary	VGP	-	wt	Q61L
WM3211	M/74	Primary	RGP	SSM	wt	wt

^a^ Tumour type of melanoma from which the cell line was developed; ^b^ VGP: vertical growth phase; RGP: radial growth phase; ^c^ NM: nodular melanoma; SSM: superficial malignant melanoma; V: valine; E: glutamic acid; Q: glutamine; L: leucine; ^p1,p2^ primary tumour-derived cell line with a metastatic pair from the same patient; ^m1,m2^ metastatic pair of the primary-derived cell line; M: male; F: female.

**Table 2:** Top 10 differentially expressed genes in resistant melanoma cell lines.

Upregulated Genes			Downregulated Genes		
Gene Symbol	Fold Change	*p*-Value	Gene Symbol	Fold Change	*p*-Value
*CXCL12*	73.055	0.030	*DMRT2*	−36.360	0.004
*COL5A1*	45.342	0.009	*MRGPRX4*	−26.686	0.002
*ALPK2*	37.803	0.005	*TRIM51*	−23.160	0.003
*ABCC3*	22.176	0.005	*CTD-2207A17.1*	−23.002	0.049
*CHST15*	21.430	0.021	*RP4-718J7.4*	−21.898	0.018
*RP11-326A19.5*	21.394	0.033	*VEPH1*	−20.866	0.000
*LAMA5*	21.264	0.010	*RP11-459E5.1*	−20.773	0.011
*SAMD11*	20.976	0.003	*GJB1*	−20.765	0.028
*RP11-54O7.3*	20.856	0.004	*ART3*	−20.169	0.015
*HHIPL2*	20.509	0.004	*FABP7*	−19.772	0.022

**Table 3:** Gene sets correlated with the resistant phenotype.

Name	ES	*p*-Value
REGULATION OF CELL POPULATION PROLIFERATION ^a^	0.219	0.013
BIOLOGICAL ADHESION ^a^	0.190	0.027
APOPTOTIC PROCESS ^a^	0.205	0.027
REGULATION OF CELL DEATH ^a^	0.204	0.030
RESPONSE TO DRUG ^a^	0.408	0.002
RESPONSE TO OXYGEN LEVELS ^a^	0.367	0.008
VASCULATURE DEVELOPMENT ^a^	0.251	0.010
PROTEIN KINASE ACTIVITY ^a^	0.350	0.005
REGULATION OF CELL DEVELOPMENT ^b^	−0.202	0.016
REGULATION OF CHROMOSOME ORGANIZATION ^b^	−0.343	0.035
REGENERATION ^b^	−0.365	0.039
GLIAL CELL DIFFERENTIATION ^b^	−0.304	0.049

ES—Enrichment score, ^a^—positive correlation (light grey rows), ^b^—negative correlation (dark grey rows).

**Table 4:** Molecular pathways associated with differentially expressed genes.

Molecular Pathway.	*p*-Value	Genes Included
ATF-2 transcription factor network ^a^	1.71E-06	*JDP2*, *MMP2*, *FOS*, *ATF3*, *SOCS3*, *JUN*, *JUNB*, *JUND*, *DUSP1*, *DUSP8*
Ensemble of genes encoding the extracellular matrix and extracellular matrix-associated proteins ^a^	3.14E-06	*A2M*, *FSTL3*, *ADAM8*, *CCL26*, *SVEP1*, *FBLN1*, *SPON2*, *AGT*, *MMP2*, *MMP11*, *VEGFD*, *LTBP4*, *CXCL12*, *AMBP*, *NID2*, *SEMA4B*, *SEMA3C*, *SFRP4*, *SEMA4G*, *POSTN*, *MUC1*, *BDNF*, *C1QL1*, *MMP24*, *GPC1*, *NTF4*, *ARTN*, *MMP25*, *COL18A1*, *OVGP1*, *PCSK6*, *ANXA8L1*, *WNT4*, *NTN5*, *COL1A1*, *COL5A1*, *WNT6*, *TSKU*, *CSF1*, *IGFBP5*, *IGFBP6*, *SRGN*, *EGLN3*, *PLXDC1*, *LAMA5*, *HTRA3*, *EDIL3*, *LGALS9*, *MMRN2*
AP-1 transcription factor network ^a^	8.49E-06	*AGT*, *FOS*, *FOSB*, *ATF3*, *HLA-A*, *JUN*, *JUNB*, *JUND*, *BCL2L11*, *DUSP1*
LPA receptor-mediated events ^a^	2.01E-04	*MAPT*, *MMP2*, *FOS*, *SRC*, *LPAR2*, *SLC9A3R2*, *JUN*, *LPAR1*
Epithelial mesenchymal transition ^a^	6.13E-04	*IL32*, *OXTR*, *JUN*, *TAGLN*, *POSTN*, *NNMT*, *MAGEE1*, *GADD45B*, *BDNF*, *MMP2*, *TPM1*, *FBLN1*, *NID2*, *MYLK*, *FSTL3*, *COL1A1*, *SFRP4*, *CXCL12*, *COL5A1*, *BASP1*, *GPC1*, *EDIL3*
Reactive oxygen species pathway ^a^	1.18E-02	*PDLIM1*, *G6PD*, *ABCC1*, *GPX3*, *TXNRD1*, *JUNB*, *FTL*
TNF-alpha signalling via NF-kB ^a^	1.46E-02	*JUN*, *SMAD3*, *GADD45B*, *CSF1*, *CEBPD*, *DUSP1*, *TNFRSF9*, *FOS*, *SOCS3*, *ZFP36*, *NFIL3*, *NINJ1*, *BCL3*, *FOSB*, *MAP3K8*, *JUNB*, *ATF3*
KRAS signalling up ^b^	6.06E-04	*ST6GAL1*, *MAP3K1*, *CCL20*, *IL10RA*, *RGS16*, *LIF*, *EMP1*, *PLAT*, *ETV1*, *ETV4*, *DUSP6*, *ETV5*, *PCSK1N*, *TSPAN13*, *C3AR1*
IL-2/STAT5 signalling ^b^	1.68E-03	*CD83*, *S100A1*, *IL10RA*, *RGS16*, *LIF*, *EMP1*, *ETV4*, *TIAM1*, *MAFF*, *BCL2*, *CTLA4*, *ITGA6*, *ICOS*, *SMPDL3A*
TNF-alpha signalling via NF-kB ^b^	1.20E-02	*DUSP4*, *NR4A1*, *DUSP2*, *CD83*, *NR4A3*, *BCL2A1*, *CCL20*, *SPHK1*, *MAFF*, *LIF*, *TNC*, *PHLDA2*
Coagulation ^b^	1.69E-02	*ACOX2*, *S100A1*, *MAFF*, *S100A13*, *PLAT*, *CTSE*, *DUSP6*, *MBL2*, *CPN1*
Early oestrogen response ^b^	2.78E-02	*TIAM1*, *MREG*, *INPP5F*, *ELOVL2*, *MYB*, *TFF3*, *BCL2*, *HR*, *RAB17*, *NBL1*, *SLC19A2*

^a^—molecular pathways linked to upregulated genes; ^b^—molecular pathways linked to downregulated genes.

## Data Availability

The data discussed in this publication have been deposited in NCBI’s Gene Expression Omnibus (Edgar R, Domrachev M, Lash AE. Gene Expression Omnibus: NCBI gene expression and hybridization array data repository Nucleic Acids Res. 2002; 30(1): 207–210) and are accessible through GEO Series accession number GSE186108 (https://www.ncbi.nlm.nih.gov/geo/query/acc.cgi?acc=GSE186108; accessed on 9 September 2021).

## Supplementary Materials

The following are available online at https://www.mdpi.com/article/10.3390/cancers13236058/s1: Figure S1: Protein expression changes after a drug holiday in the resistant melanoma cell lines. Table S1: Primer sequences for the validation of RNA seq data using RT-qPCR. Table S2: List of differentially expressed genes between BRAFi/MEKi-sensitive and -resistant melanoma cell lines. Table S3: List of downregulated genes expressed in BRAFi/MEKi-resistant cell lines. Only the top 30 genes are listed for each cell line. Table S4: List of overexpressed genes in BRAFi/MEKi resistant cell lines. Only the top 30 genes are listed for each cell line.
