# Supplementary figures and images for: Molecular Alterations Associated with Acquired Drug Resistance during Combined Treatment with Encorafenib and Binimetinib in Melanoma Cell Lines
Source: Cancers (Basel). 2021 Dec 1;13(23):6058. doi: 10.3390/cancers13236058 (PMC8656772; doi:10.3390/cancers13236058)

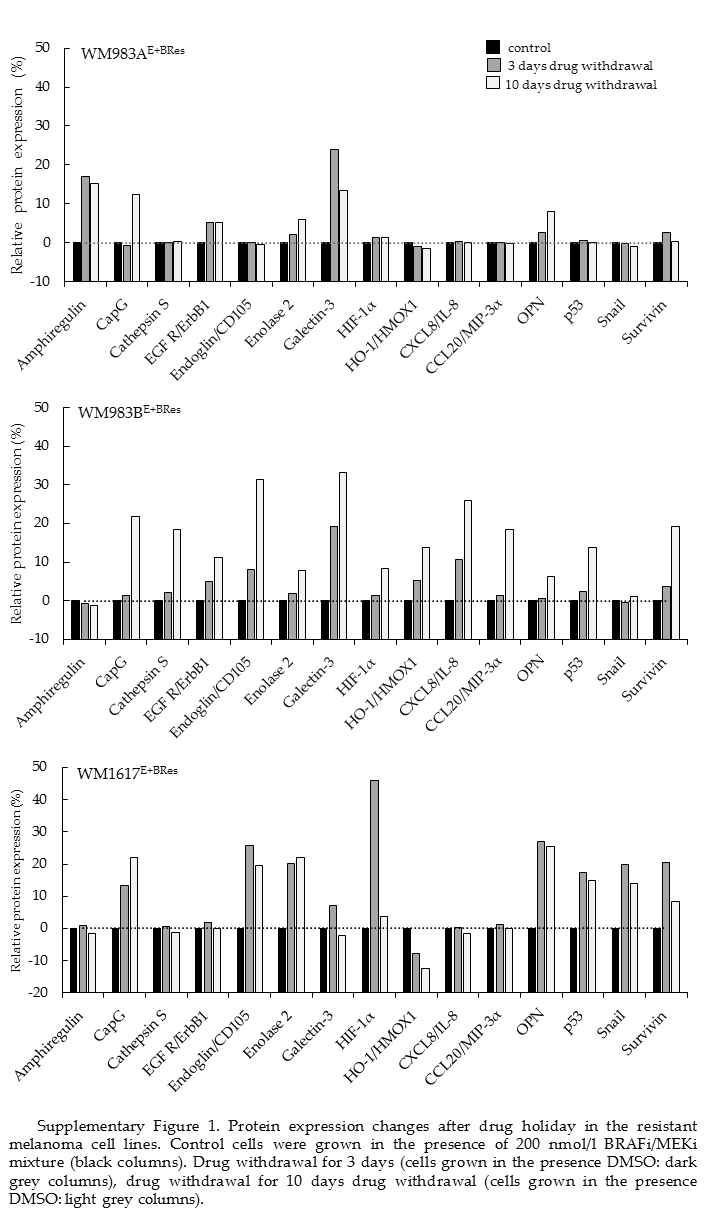

Supplement: Supplementary file 1 [file cancers-13-06058-s001.zip › Supplementary_Figure S1.tif]
